# Electrophysiological and pharmacological evaluation of the nicotinic cholinergic system in chagasic rats

**DOI:** 10.1186/2050-6511-14-2

**Published:** 2013-01-07

**Authors:** Rafael Bonfante-Cabarcas, Erlymar López Hincapié, Eliezer Jiménez Hernández, Ruth Fonseca Zambrano, Lady Ferrer Mancini, Marcos Durand Mena, Claudina Rodríguez-Bonfante

**Affiliations:** 1 Biochemistry Research Units, Health Sciences School, Universidad Centro Occidental Lisandro Alvarado, Barquisimeto, Lara, Venezuela; 2 Medical Parasitology Research Units, Health Sciences School, Universidad Centro Occidental Lisandro Alvarado, Barquisimeto, Lara, Venezuela; 3 Libertador Av. con Andrés Bello, Unidad de Bioquímica, Decanato de Ciencias de la Salud, Universidad Centro-Occidental “Lisandro Alvarado”, Barquisimeto, Estado Lara, Código Postal: 3001, Venezuela

**Keywords:** Vagal stimulation, Isolated beating hearts, Nicotine, Chagas disease, Mecamylamine, DHβE, α-BGT

## Abstract

**Background:**

Two theories attempt to explain the changes observed in the nicotinic acetylcholine receptors (nAChRs) in chagasic cardiomyopathy. The neurogenic theory proposes that receptor changes are due to loss of intracardiac ganglia parasympathetic neurons. The immunogenic theory proposes that the nAChRs changes are the result of autoantibodies against these receptors. Both theories agreed that nAChRs functional expression could be impaired in Chagas disease.

**Methods:**

We evaluated nAChRs functional integrity in 54 Sprague Dawley rats, divided in two groups: healthy and chronic chagasic rats. Rats were subjected to electrocardiographic studies in the whole animal under pentobarbital anesthesia, by isolation and stimulation of vagus nerves and in isolated beating hearts (Langendorff’s preparation).

**Results:**

Nicotine, 10 μM, induced a significant bradycardia in both groups. However, rats that had previously received reserpine did not respond to nicotine stimulation. β-adrenergic stimulation, followed by nicotine treatment, induced tachycardia in chagasic rats; while inducing bradycardia in healthy rats. Bilateral vagus nerve stimulation induced a significantly higher level of bradycardia in healthy rats, compared to chagasic rats; physostigmine potentiated the bradycardic response to vagal stimulation in both experimental groups. Electric stimulation (e.g., ≥ 2 Hz), in the presence of physostigmine, produced a comparable vagal response in both groups. In isolated beating-heart preparations 1 μM nicotine induced sustained bradycardia in healthy hearts while inducing tachycardia in chagasic hearts. Higher nicotine doses (e.g.,10 – 100 uM) promoted the characteristic biphasic response (i.e., bradycardia followed by tachycardia) in both groups. 10 nM DHβE antagonized the effect of 10 μM nicotine, unmasking the cholinergic bradycardic effect in healthy rats only. 1 nM α-BGT alone induced bradycardia in healthy hearts but antagonized the 10 μM nicotine-induced tachycardia in chagasic rats. In healthy but not in chagasic hearts, 10 μM nicotine shortened PQ and PR interval, an effect counteracted by MA, DHβE and αBGT

**Conclusion:**

Our results suggest that cholinergic function is impaired in chronic Chagas disease in rats, a phenomena that could be related to alteration on the nAChR expression.

## Background

Chagas disease, caused by *Trypanosome cruzi* (*T cruzi*), is considered a serious public health problem in Central and South America countries [1]. In Venezuela, approximately 4 million people are at risk to develop Chagas disease [2]. The chagasic chronic cardiomyopathy (CCC) is the most common complication of this disease; approximately 25-30% of infected patients developed CCC [1].

Although the CCC pathogenesis is not completely understood, two theories attempt to explain it: the neurogenic theory postulates that CCC is the result of myocardial denervation. During the acute phase of the disease, *T cruzi’s* invasion of the myocardium results in a selective, mechanical destruction of the intracardiac postganglionic parasympathetic neurons. The destruction of the parasympathetic neurons allows for a sustained non-counteracted sympathetic tone; this unbalanced sympathetic stimulation initiates trophic changes that result in the myocardial remodeling that culminates in arrhythmias and heart failure [3].

The immunogenic theory explains the CCC pathogenesis as the result of an aberrant immune response that includes loss of self-tolerance and the development of cross-reacting antibodies. Due to molecular mimicry, cross-reacting antibodies bind and neutralize surface receptors such as nAChRs. These cross-reacting antibodies (i.e., autoantibodies) affect the activity and number of those receptors population [4-7].

Nicotinic acetylcholine receptors (nAChRs) are pentameric, ligand-gated ion channels, formed by α and β subunits. Eight α subunits (α_2_- α_7_, α_9_, α_10_) and three β subunits (β_2_-β_4_) have been described; the combination of these subunits produces a wide variety of functional receptors [8]. Intracardiac ganglion neurons can express α_2_ to α_9_ and β_2_ to β_4_ subunits, assembled predominantly as α_3_/β_2_, α_3_/β_4_, α_3_/β_2_/β_4_, α_3_/β_2_/α_5_, α_3_/β_4_/α_5_, and monomeric α_7_. For example, the canine intracardiac ganglion expresses predominately α_3_/β_2_ nAChRs, with a smaller levels of α7 nAChRs [9-12].

Both neurogenic and immunogenic theories propose alterations in the function of the cholinergic system. Recently, our group demonstrated the existence of trophic and functional disturbances of the muscarinic cholinergic receptor system on *in vivo* and *in vitro* rats’ models of Chagas disease [13,14]. In the present study, we analyzed the functionality of the nAChRs in the whole animal and in isolated beating-heart preparations, of healthy and chagasic Sprague Dawley rats.

## Methods

All drugs and chemicals were purchased from Sigma-Aldrich Co (St. Louis, MO, USA) and prepared as mg/ml or M stocks solution by dissolving the drugs in purified deionized water. Stocks solutions were alliquoted and stored at 5°C until use.

### Animal model

All experiments were carried out on 54 Sprague Dawley rats, randomly distributed according to the experiment: 16 (8 healthy and 8 chagasic rats) for the whole animal experiments, 15 (8 healthy and 7 chagasic) were treated with reserpine, and 23 (11 healthy and 12 chagasic) were used in vagal stimulation and isolated beating heart experiments. The MHOM/VE/92/2-92-YBM trypomastigotes strain was used to induce infection. Experimental animal (i.e., rats) were inoculated with 1.000 trypomastigotes per gram of body weight (chagasic group). Chagasic animals develop an acute disease with a parasitemia peak of 67.27 ± 25.05 × 10^6^ parasites/ml at the third week of infection. At the time of performing the experiments, the animals had 7.84 ± 0.45 months-old, weighted 504.9 ± 10.74 and 418.5 ± 15.10 grs for healthy and chagasic rats, respectively; and only two chagasic animals displayed parasites in a blood sample, giving a parasitemia of 114.9 ± 84.43 parasites/ml. Animals were individually housed in a temperature-controlled environment with a 12:12 light/dark cycle and free access to food and water. Experimental protocols were approved by the ethical committee of the School of Health Sciences following the American Physiological Society guidelines.

### Vagus nerve stimulation (VNS)

The animals were anesthetized using a pentobarbital (20-40 mg/Kg) and ketamine (50 mg/Kg) cocktail administered intraperitoneally. The animals’ respiration was mechanically aided through a tracheal cannula connected to a volume-controlled rodent respirator at a frequency of 70 strokes/min to facilitate ventilation in spontaneously breathing rat. The cervical vagus nerve was exposed bilaterally and severed at the caudal terminus. Platinum bipolar electrodes were attached to the nerves ending leading toward the heart. The electrodes were connected to a PowerLab/8sp system to generate frequency of heart pacing . During the experiments performance, the electric pulses were modified according to the protocol. Impulses were delivered either at a fixed frequency (1.5 Hz) but different potency ranges (0.25 to 3 V) or in a range of frequencies (1-4 Hz) but fixed potency (2 V). All experiments were performed in the absence and presence of 0.3 mg/Kg physostigmine.

### Isolated beating-heart system

The animals were anesthetized as described above and the hearts removed under aseptic conditions. The isolated hearts were connected to a Langendorff’s perfusion system by cannulation of the aorta. The hearts were perfused with a tepid (37°C) modified physiological solution (pH 7.40 ± 0.05), aerated with a 95% O_2_ and 5% CO_2_ mixture. Perfusion was conducted at a rate of 7-10 mL/min maintaining a pressure range of 50 to 100 mmHg. The perfusion solution composition included 10 mM glucose, 1 mM MgSO_4_, 116 mM NaCl, 18 mM NaHCO_3_, 2.5 mM CaCl_2_, 5 mM KCl, and 1 mM malate.

To evaluate the effect of nicotine stimulation on the chagasic and control hearts’ rate, the isolated hearts were perfused, for 5 minutes, with 1, 10 or 100 μM of nicotine. The heart preparations were allowed a 10 min rest period between doses – maintaining perfusion with modified physiological solution. The effect of the following nAChRs’ antagonists, on the isolated hearts’ rate, was evaluated: 1 μM mecamylamine (MA, α_3_/β_4_ nAChR antagonist), 10 nM dihydro-β-erythroidine (DHβE; α_4_/β_2_ nAChR antagonist), and 1 nM α-bungarotoxin (α-BGT; α_7_ nAChR antagonist) [15]. The antagonists were administered in the perfusion solution for 10 minutes, in the absence of nicotine, and for additionally 5 minutes in the presence of 10 μM nicotine. The preparations were allowed a 10 minutes resting period – perfusion with modified physiological solution – between antagonists administration.

### EKG recording

In the VNS experiments, the hearts’ electric activity was monitored using needle electrodes placed subcutaneously on the sternum xiphoid process and on both shoulders − the left shoulder electrode served as reference electrode. In the isolated heart preparations, the positive electrode was inserted into the heart’s apex and the negative electrode into the right atrium. Analogical EKG signals were amplified using BioAmp, transformed in digital signals by Power Lab 8 data acquisition unit, recorded and analyzed using Lab Chart software (ADInstruments).

### Data analysis

Data are expressed either as mean of absolute values ± SEM or normalized to be expresed as percentages ± SEM. Paired and non-paired Student’s t-test were used to analyze the effect of a drug on a particular group in matched observations or when a variable for the control group was compared with the same variable of the *T cruzi* infected group, respectively. Repeated measure analysis of variance (rANOVA) followed by a Dunnet’s post-test were peformed to determine the statistical significance of drug concentration and time effect per group. In all analyses, a p value < 0.05 was considered statistically significant. Statistical analysis were performed using the GraphPad Prism 4 for Windows software (GraphPad Software Inc, La Jolla, CA).

## Results

### EKG study in intact animals

**Figure 1:**
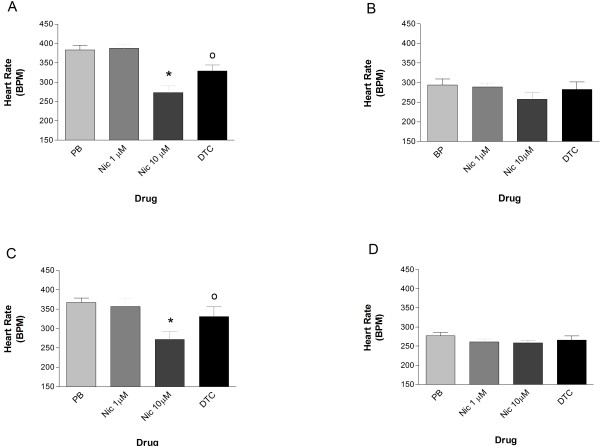
**Nicotine affects the heart rate of anesthetized whole-animals.** 10 μM Nicotine (Nic) induced a significant bradycardic response, while 1 μM D-tubocurarine reversed it, in both healthy (**A**) and chagasic (**C**) rats. Lower doses of Nic (i.e., 1 μM) have no effect on the heart rate of these animals. EKG results from healthy (**B**) and chagasic (**D**) rats, treated daily with 1 mg/Kg of reserpine for 3 days, showed a significant decrease of heart rate compared to untreated animals; however, 10 μM of nicotine has no bradycardic effect on reserpine-treated healthy (**B**) or chagasic (**C**) animals.

The significant bradycardia induced by 10 μM nicotine, in healthy and chagasic rats, was reverted by 0.1 μM d-tubocurarine (Figures 1A and 1C). Lower concentrations of nicotine appear to have no effect on the heart rate of the animals.

In order to determine whether the catecholaminergic neurons were involved in the bradycardic response, the synaptic amine content was depleted with reserpine (1 mg/Kg/day for three days) in both control and chagasic animals. It was observed that reserpine-treated animals (both groups) had a lower basal heart rate and nicotine was unable to induce bradycardia (Figure 1B and 1D).

**Figure 2:**
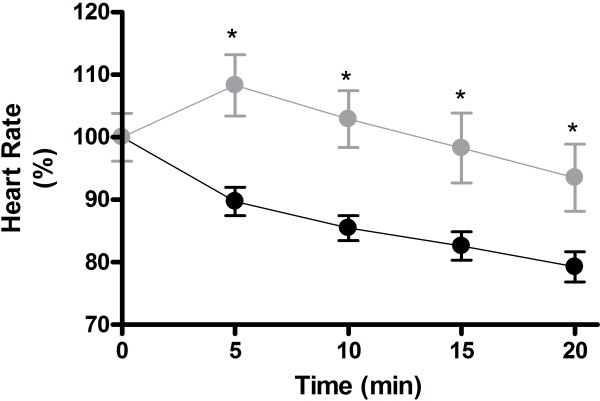
**Nicotine effect on sympathetic-stimulated whole-animals.** Anesthetized rats (n=10 per group) were treated successively with isoproterenol (0.01 mg/Kg) followed by nicotine (10 μM) at 20 min intervals. Nicotine induced bradycardia in healthy rats (black circles), while inducing tachycardia in chagasic rats (gray squares). *means p < 0.05 when both groups are compared at the indicated time.

When adrenergic tone was enhanced (0.01 mg/Kg of isoproterenol), a similar tachycardic response (p = 0.14) was induced in both animals groups (i.e., 117.2% ± 1.54 in the healthy group and 120.6% ± 1.68 in the chagasic rats). After administration of 10 uM of nicotine, we observed that nicotine induced bradycardia in the healthy group while, surprisingly, inducing a significant tachycardia in the chagasic animals (Figure 2).

### NVS study in intact animals

**Figure 3:**
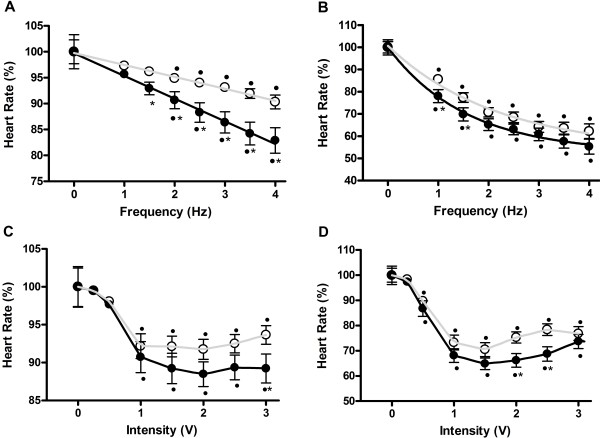
**Vagal nerve stimulation in whole-animals.** A 2 ms electric stimulation was delivered to the vagal nerve − at increasing frequency but fixed voltage (2 V) or increasing voltage at a fixed frequency (1.5 Hz) − in the absence (**A** and **C**) or presence of 0.3 mg/Kg physostigmine (**B** and **D**). In absence of physostigmine chagasic rats (open circles) the heart rate decreased proportionally to stimulus frequency (panel **A**) or intensity (panel **C**). Physostigmine increased the vagal response in both groups; however, when higher stimulation frequencies were applied(i.e., ≥ 3 Hz), the vagal response of chagasic animals became similar as compared with healthy rats. p < 0.05 indicated statistical significant differences between groups (*) or to the basal rate (^·^).

Stimulation of both vagus nerves induced a proportional bradycardia to the frequency of the stimulus. However, the bradycardic response was significantly higher in healthy rats, compared with chagasic rats (Figure 3A). Physostigmine potentiated the bradycardic response, in both experimental groups; at low frequencies (1 and 1.5 Hz) the bradycardic response was significantly higher in healthy rats but, a higher frequencies (2 to 4 Hz) bradycardia was similar in both groups (Figure 3B).

Likewise, at low frequency stimulation (1.5 Hz), a significant bradycardic response was elicited as the voltage intensity increased above 0.5 V. A significant difference between the groups was observed at 3 V when the vagus nerves’ data were analyzed together (Figure 3C). Physostigmine potentiated the bradycardic response, with the resulting response significantly higher, at 2 and 2.5 V, for healthy rats compared with chagasic rats (Figure 3D).

### Isolated beating-hearts study

#### Heart rate

**Figure 4:**
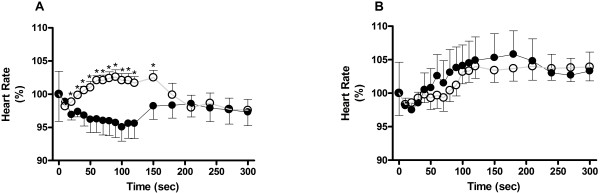
**Nicotine induces a biphasic response in isolated beating-heart preparations.** Nicotine at concentrations of 1 μM (left panel) and 10 μM (right panel) was perfused during 5 min. The bipolar 400 Hz acquisition rate EKG records indicated that 1 μM nicotine increased the heart rate of chagasic hearts (open circles), while decreasing it in healthy hearts (filled circles). 10 μM nicotine induced a biphasic effect (i.e., transiently bradychardia followed by sustained tachycardia) on both healthy and chagasic animals’ heart rate. * p < 0.05 indicated statistically significant difference compared to pre-drug basal rate.

Figure 4 illustrates the effect of nicotine on the heart rate of healthy and chagasic hearts. Nicotine (1 μM) slow-down the heart rate for 150 sec in healthy hearts (-4.9 ± 2.1%; p < 0.05), while in the chagasic hearts induced a transient but non-significant, decrease on the hearts rate (20 s), followed by a significant tachycardia (+2.6 ± 1%, p <0.05) (see Figure 4A). The response of healthy and chagasic hearts to 10 μM nicotine stimulation (Figure 4B) was comparable to that induced by 1 μM nicotine in the chagasic hearts. Initially, 10 μM nicotine induced a non-significant bradycardia (-2 ± 1.5%, p> 0.05) that was followed by a significant tachycardia (+5.8 ± 3.5% in healthy group; +4 ± 1.9% in chagasic group. p <0.05). The effect of 100 μM nicotine was similar to that induced by 10 μM nicotine in both groups.

In the absence of nicotine stimulation, healthy hearts response to MA was a slight but significant (p< 0.05) bradycardia (2.24 to 2.92%). Chagasic hearts response to MA was non-significant. However, 1 μM MA abrogated the tachycardia elicited by 10 μM nicotine, in both, healthy and chagasic hearts.

**Figure 5:**
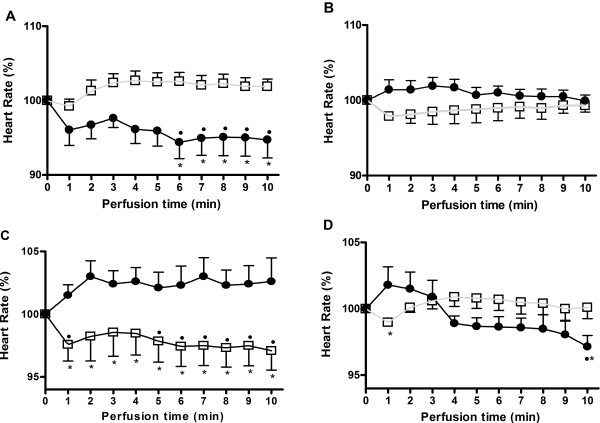
**α-BGT and DHβE pharmacological effects on isolated beating-heart preparations.** The nicotinic antagonists α-BGT (panels **A** and **B**) and DHβE (panels **C** and **D**) were perfused in the absence (black circles) or presence (open squares) of 10 μM nicotine in healthy (panels **A** and **C**) and chagasic (panel **B** and **D**) hearts. Observe that α-BGT or DHβE, in healthy hearts, induced a significant bradycardia that counteracted the nicotine effect. p < 0.05 indicates statistically significant difference between drug’s group (*) or to the basal heart rate (·).

Figure 5A shows that on healthy hearts α-BGT (full circles) induced a significant bradycardia (-6.8 ± 3.1%; p <0.05); however, in the presence of 10 μM nicotine (open squares) α-BGT blocked the nicotine-induced tachycardia. Figure 5B shows that the α-BGT, by itself, had no effect on chagasic hearts’ heart rate (full circles), but effectively blocked the nicotine-induced tachycardia (open squares).

In healthy hearts 10 nM DHβE, by itself, failed to induce a significant tachycardia (p > 0.05); however, in the presence of nicotine (10 μM), DHβE induced a significant (p < 0.05) bradycardia (Figure 5C). The chagasic hearts response to DHβE stimulation alone was a delayed bradycardia (i.e., 10 min after stimulation). In the presence of nicotine, the delayed was abrogated (Figure 5D) and the response was comparable to that observed in the healthy hearts group.

#### PQ and PR intervals

Chagasic hearts had significantly prolonged PQ and PR intervals when compared to healthy hearts (healthy Rats: PQ = 46.82 ± 2.49 and PR: 53.70 ± 2.37 ms; chagasic rats: PQ = 54.17 ± 2.55 and PR = 61.92 ± 2.48 ms; p = 0.049 and 0.041, respectively). The PQ interval in healthy hearts was significantly decreased by nicotine 10 μM, during the desensitized period (5 min), an effect that was antagonized by MA, DHβE and α-BGT. No significant effect in chagasic hearts in the PQ interval was observed. Similar effects were observed for the PR intervals. When healthy and chagasic hearts were compared in a particular protocol variable, we observed significant differences with 10 μM nicotine during the desensitizing period, 100 μM nicotine during the activation period, DHβE and αBGT (Table 1).

**Table 1:** Effect of nicotine and a selective nicotinic antagonist on electrocardiographic parameters in isolated beating heart

**Protocol Sequence**	**PQ interval**				**Aorta Pressure Wave**			
	**Healthy Hearts**		**Chagasic Hearts**		**Healthy Hearts**		**Chagasic Hearts**	
	**AV (msec)**	**%**	**AV (msec)**	**%**	**AV (mmHg)**	**%**	**AV (mmHg)**	**%**
**Basal**	46.8±2.5	100	**54.2±2.6**^**@**^	100	14.1±2.3	100	14.4±1.4	100
**Nic 1 μM A**	44.9±1.7	97.2±3.0	53.0±2.5	96.2±0.3	15.8±2.6	141.0±29.72	15.6±2.2	110.7±11.2
**Nic 1 μM D**	47.1±2.4	101.7±4.3	53.3±3.0	95.7±1.3	13.6±1.9	124.7±28.72	13.3±1.5	96.0±10.0
**Wash 1**	49.9±1.9	100	53.7±2.2	100	11.6±1.6	100	12.9±1.5	100
**Nic 10 μM A**	48.9±2.0	98.1±2.2	53.8±2.3	100.3±1.7	16.7±1.9*	180.9±37.74	16.9±1.9*	134.8±9.8
**Nic 10 μM D**	45.4±2.2*	90.7±2.2	54.2±2.5	**104.6±2.0**^**@**^	14.6±1.6	159.5±31.96	13.6±1.3	112.8±8.7
**Wash 2**	47.9±1.9	100	55.8±2.6	100	11.8±1.2	100	12.6±1.5	100
**Nic 100 μM A**	48.3±2.0	101.0±2.4	57.1±2.5	**109.0±1.5**^**@**^	16.5±1.7*	150.4±17.21	14.5±1.5	120.1±9.7
**Nic 100 μM D**	48.7±2.0	101.4±2.9	54.5±2.6	100.5±1.2	12.8±1.6	107.1±5.75	13.4±1.5	112.7±12.6
**Wash 3**	48.1±2.1	100	51.2±2.6	100	12.9±1.4	100	13.1±1.2	100
**MA**	47.4±2.0	99.6±3.8	53.0±2.4	103.4±1.7	11.7±1.5	90.5±8.13	12.3±1.3	95.3±6.4
**MA+Nic 10 μM A**	48.2±2.3	100.3±2.1	52.7±2.05	104.3±1.1	12.5±2.3	92.7±9.64	11.4±1.3	**88.7±6.0°**
**MA+Nic 10 μM D**	48.6±2.9	**101.2±2.8°**	53.6±2.4	102.8±0.4	12.4±1.6	94.4±7.88	11.4±1.4	**87.7±6.6°**
**Wash 4**	46.6±2.4	100	53.9±2.1	100	10.3±1.5	100	11.5±1.3	100
**DHβE**	47.4±2.4	101.7±1.7	55.4±2.5	**107.8±0.5**^**@**^	12.4±2.1	121.9±15.10	10.7±1.2	95.4±3.7
**DHβE+Nic 10 μM A**	49.0±2.6	**105.5±1.8°**	55.8±2.5	104.7±0.6	11.2±1.8	**110.0±12.80°**	11.0±1.2	**98.5±4.5°**
**DHβE+Nic 10 μM D**	48.8±1.9	**105.6±2.2°**	55.6±2.3	106.8±0.7	10.4±1.7	**99.8±8.64°**	10.8±1.2	96.3±4.4
**Wash 5**	48.4±2.1	100	55.8±2.6	100	10.0±1.9	100	10.1±0.9	100
**αBGT**	50.6±2.1	104.9±2.9	55.3±2.5	**97.2±0.9**^**@**^	7.7±1.4	84.3±7.33	10.6±1.0	104.2±3.8
**αBGT+Nic 10 μM A**	50.3±1.9	**104.4±1.9°**	56.0±2.9	81.3±3.6	9.4±1.7*	**96.5±5.52°**	8.9±1.2	**99.0±5.2°**^**@**^
**αBGT+Nic 10 μM D**	49.4±1.8	102.6±2.4	57.4±2.6	106.6±1.7	10.3±2.1	105.2±12.35	9.7±1.2	**95.4±3.8**^**@**^
**Wash 6**	50.3±2.1	-	57.8±2.7	-	10.4±1.0	-	9.3±1.7	-

A: agonist activation period at 30 sec of perfusion; D: agonist desensitization period at 5 min of perfusion; AV: absolute values; Nic: nicotine; MA: mecamylamine; DHβE: dihydro-beta-erythroidine; αBGT: alfa-bungarotoxin; * means p < 0.05 when absolute values are compared against basal or wash periods by repeated measure ANOVA followed by Dunnet post-test; ° means p < 0.05 when % values are compared in the presence and absence of the antagonist by Wilconxon matched pairs test; ^@^ means p < 0.05 when % values are compared between healthy and chagasic groups by Mann-Whitney test.

#### QT and QTc intervals

No significant effects on either of the intervals were observed when healthy and chagasic hearts were compared. Furthermore, we did not observe any significant effect of nicotine on both intervals; however, in chagasic hearts with the addition of 10 μM nicotine (activation period), DHβE and α-BGT significantly increased the QTc interval when compared to nicotine only. When healthy and chagasic hearts were compared in a particular protocol variable we observed significant differences with 10 μM nicotine during the desensitizing period and DHβE.

#### T and QRS amplitude

QRS amplitude was higher in healthy hearts when compared with chagasic hearts (HR: 709 ± 103.8 μV; CH: 462.1 ± 62.53 μV; p = 0.05). In healthy hearts, 10 μM nicotine in the presence of MA and DHβE only, induced a significantly decrease of the QRS amplitude; while in chagasic hearts 1 μM nicotine and 10 μM nicotine in the presence of DHβE induced a significant decrease of QRS amplitude. When healthy and chagasic hearts were compared in a particular protocol variable we observed significant differences with 100 μM nicotine during the desensitizing period and DHβE.

No differences for the T wave amplitude were observed between healthy and chagasic hearts. In healthy and chagasic hearts nicotine 10 μM in addition to DHβE induced a significant decrease of the T amplitude during the desensitizing period. When healthy and chagasic hearts were compared in a particular protocol variable we observed significant differences with 10 μM nicotine in addition to MA during the desensitizing period.

#### Perfusion pressure

In healthy and chagasic hearts 10 μM nicotine induced a significant increases of the pressure wave amplitude during the activation period, an effect that was blocked by MA and DHβE in healthy hearts and by MA, DHβE and αBGT in chagasic hearts. The use of 100 μM nicotine also induced an increase of the pressure wave amplitude during the activation period in healthy hearts but not in chagasic ones. When healthy and chagasic hearts were compared in a particular protocol variable we observed significant differences with 10 μM nicotine in addition to αBGT during activation and desensitizing periods (Table 1).

## Discussion

This work represents the first study that evaluates the functional integrity of the nicotinic cholinergic system in rats with chronic chagasic disease, using electrophysiological tools. We were able to determine that rats with Chagas disease have a dysfunction of nicotinic cholinergic system when compared with healthy rats.

In our whole-animal model, nicotine induced bradycardia, an effect that could be mediated by the simultaneous activation of the post-synaptic autonomic neurons and inhibition of adrenergic pre-synaptic terminals innervating the heart. The inhibition of the adrenergic response most likely is mediated by M2 muscarinic AChRs. M2-mediated inhibitory effect has been demonstrated in guinea pig, where muscarinic agonists reduced norepinephrine overflow, in a concentration-dependent manner, and such effect was selectively antagonized by the M2-specific antagonist AF-DX-116 [16].

The tachycardia induced by nicotine, in the presence of isoproterenol, indicates an impairment in the vasovagal reflex in chagasic rats. Isoproterenol increases the systolic pressure, due to the increment on heart rate and ejection fraction. The damaged cholinergic parasympathetic efferents favored a post-synaptic β-adrenergic dominance over the heart rate. This observation was consistent with reports that, in rats, phenylephrine-induced bradycardia was diminished in the indeterminate phase of Chagas disease as well as in chronic chagasic cardiomyopathy [14].

Direct stimulation of the vagus nerve, in rats with chronic Chagas disease, decreased the bradycardic response as a function of stimuli frequency and intensity, indicating a reduced vagal function in chagasic rats. The importance of the vagus nerve’s functional integrity has been documented in rats with acute chagasic myocarditis using direct vagal stimulation [17]. In these studies, the chronotropic response to stimulation, with low frequencies pulses, was significantly different between chagasic rats and healthy rats. These groups have comparable chronotropic response to higher frequency stimuli suggesting decrease in the fibers’ excitability and change in their response threshold in chagasic rats, due to acute nerve inflammation.

In Chagas disease, the diminished cholinergic function has been explained as a direct consequence of the presence of autoantibodies against both types of AChRs (i.e., nicotinic and muscarinic receptors). The chronic binding of these autoantibodies to the nAChR could induce a decrease in the population of functional nAChRs and consequently contribute to the alterations described in the course of chronic Chagas' disease [6,7]. In our experiments, we observed that when physostigmine, a well known acetyl cholinesterase inhibitor, was administered at the same time of high frequencies stimuli, the vagal response was restored. By mass-action law, a high level of synaptic acetylcholine would competitively displace the autoantibodies from the receptor sites.

In our isolated beating-heart model, nicotine stimulation induced the classic biphasic heart rate, which has been described for both nicotine and other non-selective AChR agonists [18-21]. However, our study demonstrated the nicotine-induced effect was dose-dependent. While 1 μM nicotine induced a bradycardia only, 100 μM of nicotine induced a biphasic effect in control rats. These differential responses were blocked by 1 μM mecamylamine, indicating that the action of nicotine used the ganglionic α3β4 nicotine acetylcholine receptor (nAChR) signaling.

The need of an intact ganglionic transmission has been demonstrated on elegant studies using hexamethonium. This ganglionic nAChR antagonist blocked the nicotine-induced biphasic heart rate [10,19-21]. However, the exact nAChR population involved in the nicotine-induced bradycardia has not been identified. Successful blockade of nicotine-induced bradycardia by α-BGT suggest that α7 nAChR subtype could be involved in the biphasic heart-rate response to nicotine stimulation [19]. Involvement of other nAChR subtypes or even the contribution of specific subunits cannot be ignored. nAChR with high affinity for nicotine are preferentially formed by α2, α4 and β2 subunits [22-24]. α2β2 and α4β2 receptors have been described to be expressed on intracardiac neurons [9]. DHβE, an α4β2 nAChR selective antagonist, prevented nicotine-induced bradycardia, while a selective agonist (RJR2403) reproduced the nicotine effect [20,21]. Cytisine, a selective β4 subunit agonist, and metillycaconitine (selective α7 antagonist) have opposites effect on the heart rate [19-21].

Our results indicated that pre-synaptic α7 nAChRs are involved in the nicotine-induced tachycardic phase as it was blocked by α-BGT. The bradycardic response to α-BGT perfused alone suggested that α7 subunit is also present in nAChR intrinsic adrenergic neurons. Recent studies have found that autonomic dysfunction; especially a decrease of vagal activity, is related to worsening of cardiovascular diseases. Autonomic imbalance with increased adrenergic and reduced parasympathetic activity is involved in the development and progress of heart failure (HF) [25]. M2-AChR knockout mice exhibit impaired ventricular function and increased susceptibility to cardiac stress, suggesting a protective role of the parasympathetic nervous system in the heart [26]. Furthermore, vagal stimulation has been shown to be beneficial in cases of heart failure, because it inhibited cardiac remodeling associated with heart dysfunction [27,28].

Our results suggest that nicotinic receptors are involved in the regulation of electrical transmission between sinusal and AV nodes, however chagasic hearts have lost this capability because nicotine was unable to shorten PQ and PR intervals in them, indicating a disregulation of nicotinic receptors in these structures. Indeed a lost of nicotinic receptors could explain a prolongued PQ and PR intervals observed in chagasic hearts in basal conditions.

The increase of perfusion pressure wave induced by nicotine in both groups reflects a positive inotropic effect of the agonist acting on nicotinic receptors. It has been already reported that nicotine produced a concentration-dependent positive inotropic effect on electrical evoked contraction of isolated toad ventricle [29].

## Conclusions

Our results support the hypothesis that cholinergic dysfunction in Chagas disease is the result of a combined disruption of the vagal transmission and trophic remodeling of intracardiac neurons and receptors. The importance of our findings is to demonstrated that alterations in cardiac nicotinic cholinergic transmission is present in Chagas disease in an early phase of cardiomyopathy evolution, before a dilated cardiomyopathy with congestive heart failure will be installed. Therefore cardiac nicotinic cholinergic functionality could be useful as prognostic marker of the disease.

## Competing interest

The authors declare that they have not competing interests.

## Authors’ contribution

RBC and CRB: made substantial contributions to conception and design, carried out whole-animal studies including data acquisition, analysis and interpretation, and wrote the draft and final version of the manuscript. ELH, RFZ and LFM: contributed to conception, design and performance of isolated beating-heart studies including data acquisition and analysis, involved in drafting the manuscript. MDM and EJM: contributed to conception, design and performance of vagal nerve stimulation studies, including data acquisition and analysis and were involved in drafting the manuscript. All authors read and approved the final version of the manuscript.

## Pre-publication history

The pre-publication history for this paper can be accessed here:

http://www.biomedcentral.com/2050-6511/14/2/prepub
